# The Effects of Increased Midsole Bending Stiffness of Sport Shoes on Muscle-Tendon Unit Shortening and Shortening Velocity: a Randomised Crossover Trial in Recreational Male Runners

**DOI:** 10.1186/s40798-020-0241-9

**Published:** 2020-02-07

**Authors:** Sasa Cigoja, Michael J. Asmussen, Colin R. Firminger, Jared R. Fletcher, W. Brent Edwards, Benno M. Nigg

**Affiliations:** 1 Human Performance Laboratory, Faculty of Kinesiology, University of Calgary, Calgary, AB Canada; 2 McCaig Institute for Bone and Joint Health, University of Calgary, Calgary, AB Canada; 3 Department of Biology, Faculty of Science & Technology, Mount Royal University, Calgary, AB Canada; 4 Biomedical Engineering Graduate Program, University of Calgary, Calgary, AB Canada; 5 Department of Health and Physical Education, Mount Royal University, Calgary, AB Canada

**Keywords:** Midsole, Bending stiffness, Running, Performance, Muscle-tendon unit, Footwear, Biomechanics

## Abstract

**Background:**

Individual compliances of the foot-shoe interface have been suggested to store and release elastic strain energy via ligamentous and tendinous structures or by increased midsole bending stiffness (MBS), compression stiffness, and resilience of running shoes. It is unknown, however, how these compliances interact with each other when the MBS of a running shoe is increased. The purpose of this study was to investigate how structures of the foot-shoe interface are influenced during running by changes to the MBS of sport shoes.

**Methods:**

A randomised crossover trial was performed, where 13 male, recreational runners ran on an instrumented treadmill at 3.5 m·s^−1^ while motion capture was used to estimate foot arch, plantar muscle-tendon unit (pMTU), and shank muscle-tendon unit (sMTU) behaviour in two conditions: (1) control shoe and (2) the same shoe with carbon fibre plates inserted to increase the MBS.

**Results:**

Running in a shoe with increased MBS resulted in less deformation of the arch (mean ± SD; stiff, 7.26 ± 1.78°; control, 8.84 ± 2.87°; *p* ≤ 0.05), reduced pMTU shortening (stiff, 4.39 ± 1.59 mm; control, 6.46 ± 1.42 mm; *p* ≤ 0.01), and lower shortening velocities of the pMTU (stiff, − 0.21 ± 0.03 m·s^−1^; control, − 0.30 ± 0.05 m·s^−1^; *p* ≤ 0.01) and sMTU (stiff, − 0.35 ± 0.08 m·s^−1^; control, − 0.45 ± 0.11 m·s^−1^; *p* ≤ 0.001) compared to a control condition. The positive and net work performed at the arch and pMTU, and the net work at the sMTU were significantly lower in the stiff compared to the control condition.

**Conclusion:**

The findings of this study showed that if a compliance of the foot-shoe interface is altered during running (e.g. by increasing the MBS of a shoe), the mechanics of other structures change as well. This could potentially affect long-distance running performance.

## Key Points

- Individual compliances of the foot-shoe interface have been suggested to store and return elastic strain energy during running via (1) ligamentous and tendinous structures or (2) by increasing the midsole bending stiffness, compression stiffness, and resilience of sport shoes. How these structures interact with each other when one of them is altered, however, is unknown.
- We showed that if one of these structures was altered (e.g. by increasing the midsole bending stiffness of a shoe), the mechanics of other compliances changed as well.
- Increasing the midsole bending stiffness of a running shoe reduced the deformation of the arch, the shortening of the plantar muscle-tendon unit, and the shortening velocities of the plantar and shank muscle-tendon units.
- This could potentially have implications on the metabolic cost of running and therefore affect long-distance running performance.

## Background

In the stance phase of running, multiple structures (e.g. running shoe, foot arch, tendons, etc.) interact with each other to transmit forces produced by the lower limb muscles through the foot to the ground. Some of these structures have been suggested to store and release elastic strain energy via ligamentous and tendinous elements [1–3] or by increased midsole bending stiffness (MBS) [4, 5], compression stiffness, or resilience [6] of a running shoe. Some of the largest elastic structures surrounding the foot-shoe interface are the Achilles tendon (AT) and the plantar aponeurosis (PA). It is commonly believed that these elastic structures store energy as they are stretched during running [7–9]. This energy is thought to be at least partially returned to the runner and used for propulsion in late stance [10]. The PA and AT energy return estimates were suggested to be approximately 3–17 [1–3] and 10–70 J/step [1, 9], respectively. In vitro measurements performed by Ker et al. [1] suggested that 17% of the total lower limb mechanical work can be returned by the foot arch in the form of elastic strain energy. More interestingly, it was shown that if the stiffness of the arch was reduced (e.g. by cutting passive elastic structures), its energy return properties decreased [1]. More recent in vivo experiments, however, showed that these values were likely overestimated, and that the arch can return only up to 8% of the total lower limb mechanical work [2]. Furthermore, studies suggested that compared to barefoot running, wearing footwear may limit arch deformation and therefore alter the stiffness of the arch, potentially affecting the spring-like function of the foot [11]. Because this previous work compared barefoot versus shod running only, it remains unclear if these findings are related to wearing footwear, in general, or to systematic differences in specific footwear features such as the MBS.

The MBS of sport shoes has been shown to have large effects on lower limb biomechanics and athletic performance [5, 12, 13]. It was suggested that the main effects of increased MBS (e.g. by placing a carbon fibre plate along the full length of a shoe) are to (1) minimise energy loss at the metatarsophalangeal (MTP) joint [6, 12], (2) store and return elastic strain energy to the foot-shoe interface [4, 5], and (3) alter the force-velocity profile of the major ankle plantarflexor muscles [14, 15]. These changes would then allow for more economical force generation [14–16] and possibly a lower energy cost of locomotion [17, 18]. In brief, the principle of minimising energy loss suggests that if less negative work is performed at a joint, muscles crossing the joint perform less eccentric work, which could result in lower energy cost of locomotion because lengthening and shortening incurs a higher energy cost compared to isometric, zero net work contractions [19]. This idea, however, is hypothetical and experimental evidence supporting the principle that minimising energy loss can be used to enhance sport performance is still missing. The principle of storing and returning elastic strain energy via resilient cushioning material of the midsole suggests that the maximum possible energy storage and return can be estimated by modelling the midsole as an idealised compressive spring [20]. Similarly, it was thought that elastic energy can be stored and returned due to longitudinal bending of the midsole, which could be modelled as an idealised torsional spring [4–6]. Previous literature addressed the principle of storing and returning energy in the midsole due to longitudinal bending, but the results are inconclusive, as some studies suggested that the carbon fibre plates are able to store and return elastic energy indicated by more positive work performed at the MTP joint [4, 5] or increased ground reaction forces (GRF) [5], whereas other studies suggested that other footwear features are the primary cause of these observed increases in positive work done at the MTP joint [6]. Lastly, the principle of optimising for muscle function suggests that the variable gearing during running (i.e. the ratio between muscle-tendon unit moment arm and GRF moment arm relative to a joint centre) [21] can be altered by changing the MBS of footwear so that muscle forces are generated at slower speeds [14, 22]. This is speculated to reduce the muscle energy cost of generating the necessary forces to execute an athletic task [14, 22, 23].

The purpose of this study was to investigate how structures of the foot-shoe interface are influenced during running by changes to the MBS of sport shoes. Specifically, the behaviour of a plantar muscle-tendon unit (pMTU), which is a representation of the PA and intrinsic foot muscles [24, 25], was studied because it spans across the full length of the foot, and therefore not only crosses the MTP but also the arch of the foot. It was hypothesised that as the MTP joint undergoes extension (i.e. dorsiflexion), and therefore negative work is performed at the joint, the pMTU will perform positive work at the arch due to the windlass mechanism [26]. Because of this mechanism, it was expected that the positive work at the arch will be reduced when running with increased MBS, as the extension of the MTP will be limited [5, 6, 12]. Furthermore, a secondary purpose of this study was to investigate the energetic behaviour of a shank muscle-tendon unit (sMTU), which is a representation of the triceps surae muscle and the AT. The behaviour of the sMTU was studied because it is a major positive work generator of the lower limb during running [27, 28], and thus corresponds to a large portion of the total metabolic cost of running [9]. It was hypothesised that running in shoes with increased MBS would result in reduced shortening velocities of the sMTU [15]. It needs to be noted that the MTU models developed in this study represent approximations of biarticulated MTUs. As such, it is possible that the extrinsic foot muscles and the knee joint orientation may affect the pMTU and sMTU mechanics, respectively. This study, however, assumed the pMTU to represent a functional unit consisting of intrinsic foot muscles and the PA, and addressed the sMTU mechanics at its distal end, only.

## Methods

### Experimental Set-up and Data Collection

#### Participants

The detailed protocol has been described previously [5]. In brief, 13 male, recreational runners (mean ± SD; height, 162.8 ± 0.5 cm; body mass, 70.5 ± 8.3 kg) performed running trials in 2 shoe conditions. All participants were moderately active, free of neuromuscular disorders and lower limb injuries in the past 6 months before participation, and fit a US men’s size 9 shoe. Also, all participants gave written informed consent prior to participating in this study.

#### Footwear Conditions

The control condition consisted of a commercially available running shoe (Nike Free 5.0, Nike Inc., Beaverton, USA), and the stiff condition was achieved by inserting straight carbon fibre plates in between the midsole and the factory insole of the control shoe. The MBS of the entire shoe was determined using a 3-point bending test [5]. Values of 1.2 N/mm and 11.9 N/mm were obtained for the control and stiff condition, respectively. The masses of the shoe conditions were determined using a laboratory balance (Model PG4002-S, Mettler-Toledo, Columbus, USA) and were 225.67 g and 289.10 g for the control and stiff condition, respectively.

#### Biomechanical Testing

For in vivo biomechanical testing, participants ran on an instrumented treadmill (Bertec Corporation, Columbus, USA) at 3.5 m·s^−1^ in both shoe conditions. The order of conditions was randomised across participants. After participants performed familiarisation trials of 10–15 s to get accustomed to the running speed and footwear conditions, the speed of the belt was increased to 3.5 m·s^−1^ and data were collected for 30 s approximately 2 s after speed was attained. This familiarisation period was deemed sufficient because all participants were experienced in treadmill running [29]. Furthermore, the fact that the footwear conditions were randomised between participants should have reduced potential confounding effects of treadmill or footwear habituation. The stance phases from 30 steps were identified and used for further analyses. Three-dimensional (3D) kinematic and kinetic data were measured using eight high-speed cameras (Motion Analysis, Santa Rosa, USA) and a single force plate instrumented in the treadmill. Twenty-five retroreflective markers were mounted on the following anatomical landmarks: distal phalanx of the great toe (GT), third toe, and fifth toe; distal heads of the first (MP1) and fifth metatarsals; navicular tuberosity (NT); medial (MH), lateral, and proximal heel; medial and lateral malleolus; proximal, distal, and posterior shank; medial and lateral epicondyles; proximal, distal, and posterior thigh; left and right greater trochanter; right and left anterior superior iliac spine; and right and left posterior superior iliac spine. For this manuscript, however, only the first 16 markers mentioned above were used for analysis. Holes were cut in the shoe to allow for the application of markers on the skin overlying the distal head of the first metatarsal, navicular tuberosity, and medial heel, as participants did not wear socks during the running trials [4]. Motion data and GRFs were recorded at 240 and 1000 Hz, respectively.

#### Dynamometry and Ultrasound Testing

A dynamometry and ultrasound session [30] was performed immediately before the biomechanical testing to estimate the moment arm of the sMTU (MA_sMTU_; i.e. Achilles tendon moment arm). For this, participants were seated in a Biodex System 3 dynamometer (Biodex Medical, Shirley, USA) and the ankle joint axis was aligned with the dynamometer axis. The foot was placed on the dynamometer foot plate and tightly secured using straps. The ankle and knee joints were oriented to 0° (neutral) and 60° (flexion), respectively. Straps were used to limit the participant’s hip and thigh motion. A 50-mm linear-array probe was placed over the myotendinous junction of the AT, which captured its trajectory at a sampling frequency of 78 Hz on a Logiq E9 ultrasound system (gain 50 dB, depth 3.0 cm, frequency 13 MHz; GE Healthcare, Chicago, USA). The AT moment arm was estimated using the tendon excursion method [31]. For the tendon excursion method to be valid, it is important that the tendon elongation can be measured where no passive moment is present [32]. Thus, the foot was rotated from 0 to 20° plantarflexion, the range over which no appreciable passive moment (< 1 Nm) was present. For this, the myotendinous junction elongation was tracked manually from the ultrasound images over the entire range of motion using ImageJ (NIH, Bethesda, USA). It needs to be noted that absolute values obtained using the tendon excursion method could be erroneous [33]. In a study of a within-subject design, however, it is assumed that this error would not differ between footwear conditions and therefore would not affect the conclusions drawn from the findings of the study.

### Data Processing and Analysis

#### MTP and Ankle Joint Kinematics and Kinetics

Raw kinematic and kinetic data were analysed using a custom written MATLAB code (Version 2019b; the MathWorks Inc., Natick, USA). Force data were downsampled to 240 Hz by performing a shape-preserving, piecewise cubic interpolation. To determine 3D MTP and ankle joint kinematics and kinetics, marker and force data were filtered using a dual pass 2nd order (i.e. zero-lag fourth order) Butterworth filter with a cut-off frequency of 50 Hz. A Newton-Euler approach was used to describe joint motion (sequence: flexion-extension, abduction-adduction, internal-external rotation), and an inverse dynamics approach was used to calculate sagittal internal joint moments to represent the moment primarily attributed to muscle forces. The MTP joint centre was estimated halfway along the MTP joint axis, which was defined by a line connecting the distal heads of the first and fifth metatarsal. The MTP joint moment was set to zero when the centre of pressure (COP) was proximal to the MTP joint axis [6, 27, 30]. The moment arm of the GRF to the MTP joint centre was determined as the perpendicular distance of the COP relative to the MTP joint axis [34]. Joint powers were calculated as the product of internal joint moment and angular velocity. Positive and negative joint work were determined as the integral of the positive and negative joint power-time curves over the stance phase, respectively.

#### Ground Reaction Force Partitioning for Multiple Foot Segments

The GRFs measured by a force plate act on a single point, the COP. During the stance phase of running, however, multiple foot segments are in contact with the ground. The different forces that act on individual foot segments cannot be determined with a single force plate. To partially overcome this limitation, a weighted probabilistic approach was used to partition the GRF for individual foot segments [24]. In brief, the magnitude of force that was partitioned for the rearfoot and midfoot depended on the vertical trajectory of each segment’s centre of mass, based on a 3D marker data relative to a global coordinate system, and its antero-posterior distance to the COP. Once the COP progressed distally to the MTP joint axis, the force acting on the rearfoot was set to zero because at these instants the rearfoot was assumed to not be in contact with the ground anymore.

#### Arch Mechanics and Muscle-Tendon Unit Models

**Fig. 1:**
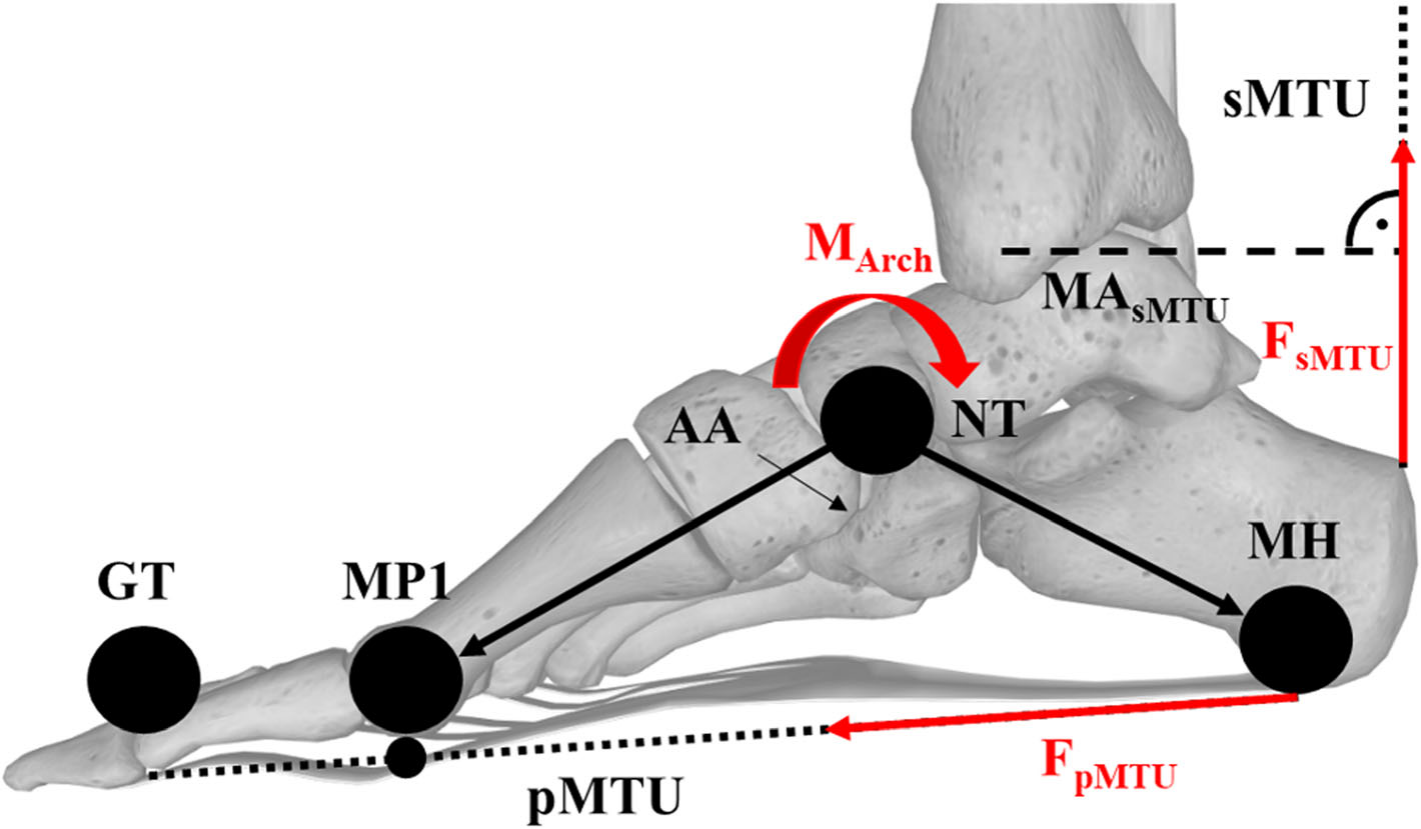
Schematic of the sagittal plane model used to estimate the arch angle (AA) using markers placed on the medial heel (MH), navicular tuberosity (NT), and distal head of the first metatarsal (MP1). The shank muscle-tendon unit (sMTU) was estimated along the orientation of the shank. sMTU force (F_sMTU_) was calculated based off a musculoskeletal model [35] and in vivo ultrasound imaging of the sMTU moment arm (MA_sMTU_). The plantar muscle-tendon unit (pMTU) was estimated spanning from MH to the great toe (GT). pMTU force (F_pMTU_) was approximated using vertical ground reaction forces and F_sMTU_ [36]. Modified from [37]

Marker trajectories that were used to describe arch and pMTU kinematics, namely MH, NT, MP1, and GT, were filtered with a cut-off frequency of 20 Hz. The mechanics of the arch were estimated during the stance phase of running by performing a sagittal plane analysis using the partitioned GRF and 3D trajectories of the MH, NT, and MP1 markers. The arch angle (AA) was determined as the 3D angle between two vectors, namely between MH and NT, and MP1 and NT (Fig. 1). Therefore, the NT was set as the centre of the arch joint. The angular velocity of the arch was determined by the first time-derivative of the angular deformation. The rear- and midfoot centre of masses were estimated halfway between the MH and NT, and MP1 and NT markers, respectively. The arch joint moment was calculated using an inverse dynamics approach, where the forces generating the moment were assumed to be the partitioned GRFs, gravity, and joint reaction forces [24].

**Fig. 2:**
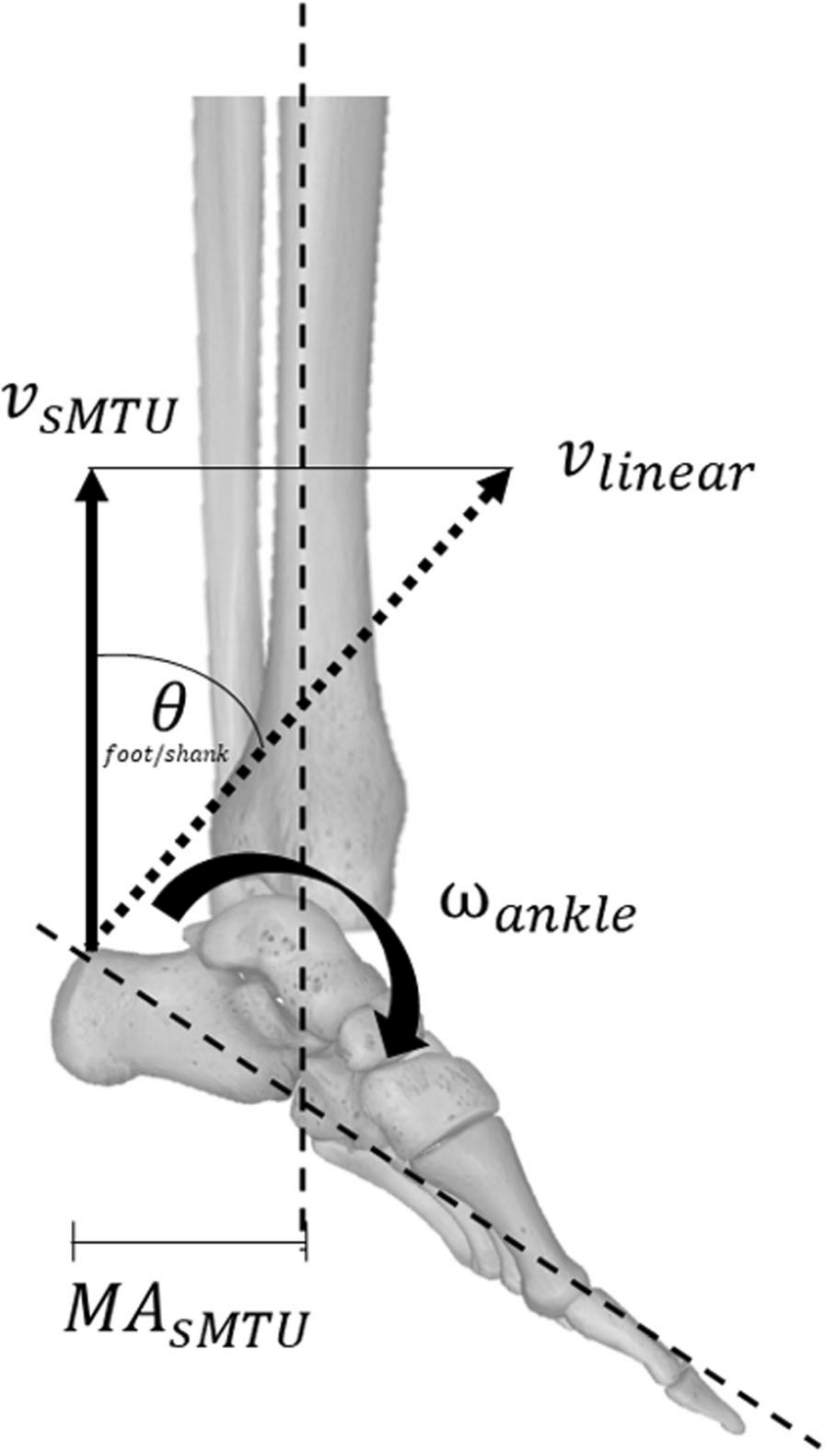
The shank muscle-tendon unit velocity (v_sMTU_) was estimated using the ankle angular velocity (ω_ankle_), the linear velocity at the proximal end of the foot (v_linear_), the shank muscle-tendon unit moment arm to the ankle joint centre (MA_sMTU_), and the angle between v_linear_ and the shank segment (θ_foot/segment_). Modified from [37]

The sMTU and its force (F_sMTU_) were estimated based on a previously described musculoskeletal model [30, 35]. For this, the sagittal plane ankle joint moment was divided by the MA_sMTU_, which was corrected for the ankle angle [38]. sMTU stretch/shortening velocity (v_sMTU_) was approximated by multiplying the ankle joint angular velocity with the MA_sMTU_ [21] (Fig. 2). This estimated the linear velocity acting on the proximal end of the foot segment, where the sMTU was assumed to be attached. This linear velocity, however, acts perpendicular to the foot segment, which is not necessarily the orientation of the sMTU, as it was assumed to be in parallel with the shank segment. For this reason, a correction was performed that accounted for the orientation of the sMTU relative to the velocity at the proximal end of the foot using:

1 $$ {v}_{\mathrm{sMTU}}={\mathrm{MA}}_{\mathrm{sMTU}}\times {\omega}_{\mathrm{ankle}}\times \cos {\theta}_{\mathrm{foot}/\mathrm{shank}} $$

where *v*_sMTU_ is the stretch/shortening velocity of the sMTU, *ω*_ankle_ is the ankle joint angular velocity, and *θ*_foot/shank_ is the angular difference between the linear velocity at the proximal end of the foot segment and the shank. The force and stretch/shortening velocity of the sMTU are therefore approximations of the mechanics at the distal end of the sMTU (i.e. AT).

The pMTU was based on a geometrical model using kinematic data of retroreflective markers placed on the MH, MP1, and GT [24, 25]. For this, the MH and GT defined the origin and insertion of the pMTU, respectively. The length of the pMTU was estimated as the sum of distances between MH and MP1, and GT and MP1. This allowed the estimation of pMTU length changes during the stance phase of running due to relative foot segment motion. MP1 acted as a tether point which the pMTU was wrapped around. If the MTP joint was extended, the tether point rotated in conjunction with the origin of the pMTU. This increased the length of the distal part of the pMTU, between MP1 and GT. Therefore, the length of the pMTU was corrected by estimating the arc length (Arc_pMTU_) due to MTP joint extension using:

2 $$ {\mathrm{Arc}}_{\mathrm{pMTU}}={r}_{\mathrm{MP}1}\times {\theta}_{\mathrm{MTP}} $$

where Arc_pMTU_ is the wrapping length of the pMTU around the tether point on MP1, *r*_MP1_ is the estimated radius of the distal head of the first metatarsal (i.e. 9.2 mm) [39], and *θ*_MTP_ is the angular deformation of the MTP joint.

shortening/shortening velocity (*v*_pMTU_) was determined by the first time derivative of the pMTU length changes. The pMTU force (*F*_pMTU_) was estimated based on cadaveric work from Cheung et al. [36] using:

3 $$ {F}_{\mathrm{pMTU}}=0.1762\times {F}_{\mathrm{vGRF}}+0.3285\times {F}_{\mathrm{sMTU}} $$

where *F*_*pMTU*_ is the force acting along the pMTU, *F*_vGRF_ is the vertical ground reaction force, and *F*_sMTU_ is the estimated AT force.

Arch and MTU power were determined as the product between arch angular velocity and joint moment, and MTU stretch/shortening velocity and force, respectively. Joint and MTU positive and negative work were determined by the time integral of the positive and negative power curves, respectively. It needs to be noted that contributions from individual compartments of MTUs (i.e. the muscle or the tendon) cannot be distinguished from each other using the methods described above.

### Statistics

Shapiro-Wilk tests were performed to test for normality of the variables of interest. These variables included (1) positive, negative, and net work; peak change in angle; take-off angle; peak moment; and peak flexion velocity at the second half of stance for the arch; (2) positive, negative, and net work; peak length change; take-off length; peak force; and peak shortening velocity during the second half of stance for the pMTU; and (3) positive, negative, and net work; peak shortening velocity; and peak force for the sMTU. If a Shapiro-Wilk test revealed a normal distribution, a paired *t* test was performed to test for significant differences between stiffness conditions; otherwise, the Wilcoxon signed-rank test was used. The significance level *α* was set to 0.05, and the Benjamini-Hochberg method was used to correct for multiple comparisons by adjusting individual *p* values [40]. Effect size estimates were calculated using Cohen’s d to aid in the interpretation of significant findings.

## Results

**Fig. 3:**
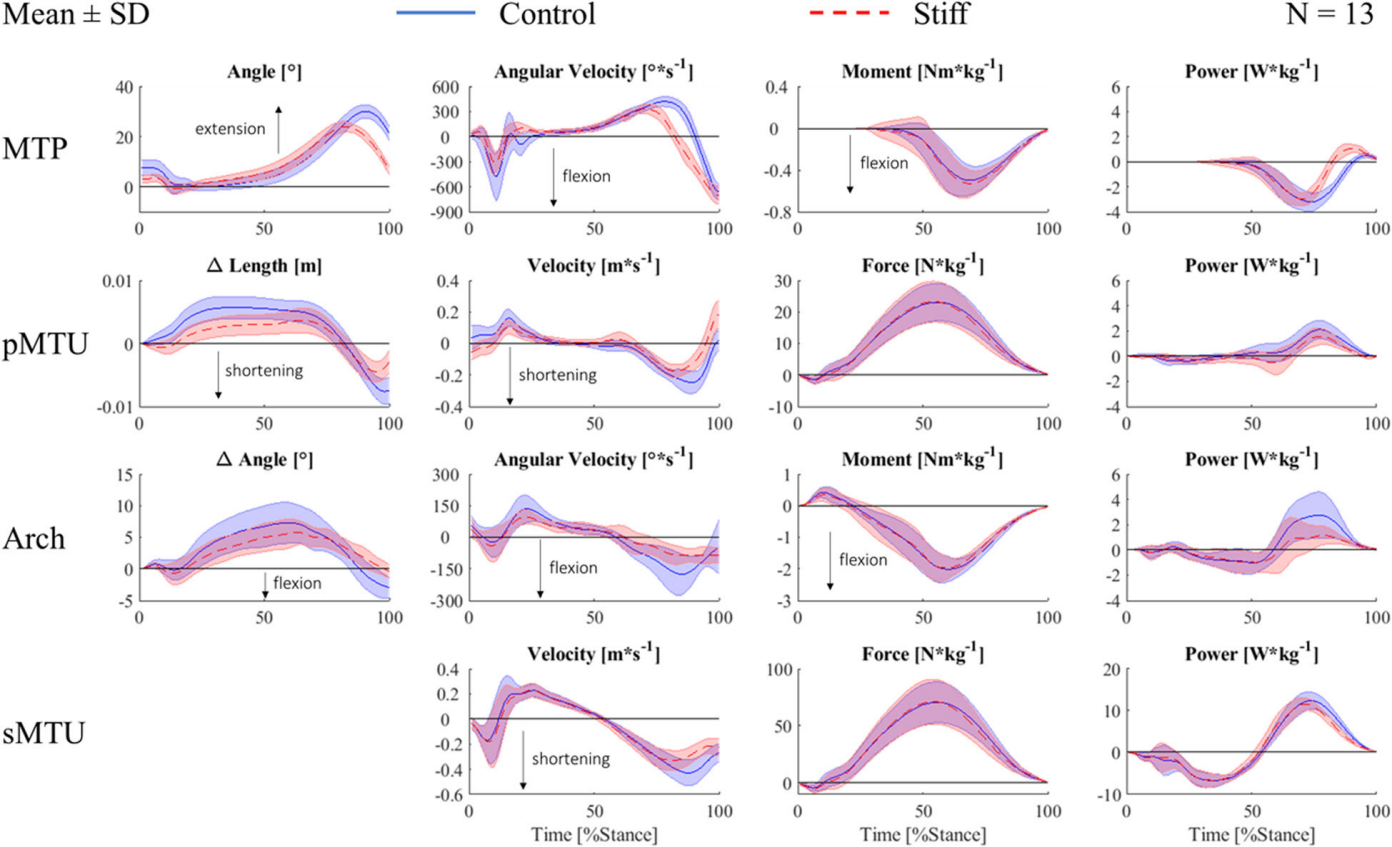
Group mean ± standard deviation (shaded area) of the metatarsophalangeal joint (MTP; first row), plantar muscle-tendon unit (pMTU; second row), arch (third row), and shank muscle-tendon unit (sMTU; fourth row) angle/length change (first column), (angular) velocity (second column), force/moment (third column), and power (fourth column) across the stance phase of running in the control (blue full line) and stiff (red broken line) conditions

The positive work done at the arch was significantly (*p* ≤ 0.001, *d* = 1.36) lower in the stiff (0.11 ± 0.05 J·kg^−1^) compared to the control (0.19 ± 0.07 J·kg^−1^) condition. There was no difference in negative arch joint work (control, − 0.10 ± 0.06 J·kg^−1^ vs. stiff, − 0.11 ± 0.06 J·kg^−1^; *p* = 0.51; *d* = 0.17). Net arch joint work was significantly (*p* ≤ 0.001, *d* = 1.11) reduced in the stiff (0.00 ± 0.09 J·kg^−1^) compared to the control (0.09 ± 0.08 J·kg^−1^) condition. The peak arch joint moment did not differ between stiffness conditions (control, − 2.10 ± 0.33 Nm·kg^−1^ vs. stiff, − 2.07 ± 0.32 Nm·kg^−1^; *p* = 0.29; *d* = 0.10). Peak arch flexion velocity during the second half of stance phase, however, was significantly (*p* ≤ 0.01, *d* = 1.57) lower in the stiff (− 186.21 ± 36.10°·s^−1^) compared to the control (− 292.78 ± 88.81°·s^−1^) condition. The peak change in arch angle relative to the angle at heel-strike was significantly (*p* ≤ 0.05, *d* = 0.66) lower in the stiff (7.26 ± 1.78°) compared to the control (8.84 ± 2.87°) condition. Also, the arch angle at take-off relative to heel-strike was significantly (*p* ≤ 0.001, *d* = 1.96) lower in the stiff (− 0.55 ± 1.01°) compared to the control (− 3.07 ± 1.51°) condition (Fig. 3).

The positive work performed by the pMTU was significantly (*p* ≤ 0.001, *d* = 1.11) lower in the stiff (0.09 ± 0.03 J·kg^−1^) compared to the control (0.13 ± 0.04 J·kg^−1^) condition. There was more negative work (*p* ≤ 0.001, *d* = 0.84) done by the pMTU in the stiff (− 0.06 ± 0.03 J·kg^−1^) compared to the control (− 0.04 ± 0.02 J·kg^−1^) condition. The net pMTU work was significantly (*p* ≤ 0.001, *d* = 1.47) lower in the stiff (0.03 ± 0.04 J·kg^−1^) compared to the control (0.10 ± 0.05 J·kg^−1^) condition. There was no significant difference in peak pMTU force between stiffness conditions (control, 23.30 ± 5.95 N·kg^−1^ vs. stiff, 23.48 ± 6.50 N·kg^−1^; *p* = 0.59; *d* = 0.03). Peak pMTU shortening velocity during the second half of stance, however, was significantly (*p* ≤ 0.001, *d* = 2.09) lower in the stiff (− 0.21 ± 0.03 m·s^−1^) compared to the control (− 0.30 ± 0.05 m·s^−1^) condition. Peak pMTU length change relative to heel-strike was significantly (*p* ≤ 0.01, *d* = 1.37) smaller in the stiff (4.39 ± 1.59 mm) compared to the control (6.46 ± 1.42 mm) condition. Also, the pMTU length at take-off relative to heel-strike was significantly (*p* ≤ 0.001, *d* = 2.41) less shortened in the stiff (− 2.30 ± 1.98 mm) compared to the control (− 7.25 ± 2.13 mm) condition.

There was no difference in positive (*p* = 0.40, *d* = 0.24) or negative (*p* = 0.39, *d* = 0.01) work performed by the sMTU between stiffness conditions. The positive and negative work were 0.76 ± 0.13 J·kg^−1^ and − 0.45 ± 0.11 J·kg^−1^ in the control and 0.73 ± 0.11 J·kg^−1^ and − 0.45 ± 0.10 J·kg^−1^ in the stiff condition, respectively. The sMTU, however, performed significantly (*p* < 0.05, *d* = 0.23) less net work in the stiff (0.28 ± 0.10 J·kg^−1^) compared to the control (0.31 ± 0.14 J·kg^−1^) condition. Further, peak sMTU force did not differ between stiffness conditions (control, 71.34 ± 18.04 N·kg^−1^ vs. stiff, 71.89 ± 19.70 N·kg^−1^; *p* = 0.59; *d* = 0.03); however, peak shortening velocity was significantly (*p* ≤ 0.001, *d* = 1.07) lower in the stiff (− 0.35 ± 0.08 m·s^−1^) compared to the control (− 0.45 ± 0.11 m·s^−1^) condition.

## Discussion

The purpose of this study was to investigate how structures of the foot-shoe interface are influenced during running by changes to the MBS of sport shoes. It was hypothesised that running with increased MBS would result in less positive work performed at the arch as the windlass mechanism is limited due to reduced MTP joint extension. The findings of this study supported the first hypothesis, as the positive work performed at the arch was lower in the stiffer shoe condition. Lower positive work at the arch when running with increased MBS occurred due to reduced arch flexion velocities and pMTU shortening velocities. No differences in arch flexion moments or pMTU forces were observed between stiffness conditions. Furthermore, less arch extension and flexion, and less pMTU stretching and shortening were observed in the stiff compared to the control condition. Also, at take-off, both structures were in a more extended and lengthened position when running in the stiff condition. Running with increased MBS altered the spring-like function of the foot by reducing the deformation of the arch and the stretching and shortening of the pMTU. Maintaining the same athletic movement (i.e. treadmill running) while reducing the mechanical work contributions of MTUs by increasing the MBS of sport shoes could be indicative of more efficient locomotion.

Previous studies have proposed that running with increased MBS resulted in better athletic performance because it allowed the major ankle plantarflexor muscles (e.g. triceps surae) to generate force more economically [15, 22]. For this reason, it was hypothesised that running in the stiff condition would result in lower shortening velocities of the sMTU. The results of this study showed significantly lower shortening velocities of the sMTU in the stiff compared to the control condition. There were no differences in sMTU forces between stiffness conditions. The net work performed by the sMTU, however, was lower in the stiff condition. Reduced shortening velocities of the entire sMTU can originate from slower shortening of the muscle (i.e. triceps surae) or the tendon (i.e. AT) in series. In the first case, reduced shortening velocities of the muscle could be indicative of reduced rates of force generation. Changes in rates of force generation have been shown to be related to changes in metabolic cost of running (i.e. cost of force generation hypothesis) [41, 42]. The cost of force generation hypothesis suggests that increases in metabolic cost are inversely proportional to contact time [43]. In support of this hypothesis, the contact times in this data set were significantly increased by ~ 13 ms per step when running in the stiff compared to the control condition, as described previously [5], which represents a 4.76% decrease in rate of force generation. This reduced rate of force generation should reduce the metabolic cost of running because the triceps surae muscles can generate the same force at a slower velocity, thus reducing the level of motor unit recruitment [23]. It is plausible to speculate that a ~ 5% reduction in the triceps surae rate of force generation could contribute to subtle differences in metabolic cost of running between stiffness conditions. In the latter case, where differences in sMTU shortening velocity originated from slower tendon shortening, this could have implications for the energy return properties of the tendon. Slower tendon shortening would result in reduced positive power and therefore less returned energy by the tendon. Many studies have speculated that energy storage and return of tendinous structures are beneficial for running [7, 44]. Therefore, if running with increased MBS reduced tendon-shortening velocities, and therefore released energy, then this could be considered disadvantageous for running performance. This study, however, cannot address if the changes in sMTU shortening velocity were due to slower shortening of the muscle or the tendon. It can only address the fact that running with increased MBS has an effect on the shortening velocities of MTUs of the foot-shoe interface. Therefore, future studies should try to answer this question by using in vivo ultrasound imaging of the muscle fascicles of the sMTU (i.e. gastrocnemius medialis, gastrocnemius lateralis, or soleus muscle) or the myotendinous junction of the AT [15].

The results of this study showed that the pMTU length at take-off was shorter than at heel-strike. This is probably due to the windlass mechanism. It is hypothesised that the PA pulled the calcaneus closer to the distal metatarsal heads as the MTP joint underwent extension, and therefore, the pMTU was shortened [26, 45]. In the stiff condition, however, the peak MTP joint extension was lower, which likely limited the wrapping of the pMTU around the distal metatarsal heads, and therefore, the length at take-off was closer to its initial length at heel-strike compared to the control condition.

Although the MTP joint showed increased flexion velocities in the stiff condition, the pMTU, which crosses the MTP joint and therefore contributes to MTP joint mechanics, showed slower shortening velocities. This means that the increased MTP joint flexion velocities must be caused by some other mechanism than the behaviour of the pMTU. It is possible that the carbon fibre plates that were inserted to increase the MBS of the running shoe are related to the increases in MTP joint flexion velocities. Elastic strain energy that is stored in the carbon fibre plates as the MTP joint undergoes extension could be returned during late stance, increasing joint flexion velocities.

Kelly et al. [11] proposed that the foot-shoe interface can be modelled as two springs that act in series, where the viscoelastic midsole of a shoe and the foot arch behave like compressive springs with given stiffnesses. This model is based on the assumption that the neuromuscular system aims to maintain a constant system stiffness during locomotion [46, 47]. The findings of this study showed that if the MBS of a running shoe, which is thought to behave as a torsional spring [4–6], is increased, the deformation of linear/rotational compliances (e.g. arch, pMTU) surrounding the foot-shoe interface is reduced. The forces and moments acting on these structures, however, did not differ between footwear conditions. Therefore, if the mechanical load on these structures remained the same but the deformation was reduced, it can be concluded that the individual apparent stiffness increased. This further supports previous findings by Kelly et al. [11] that the foot-shoe interface can be modelled as multiple compliances that act in series:

4 $$ {k}_{\mathrm{foot}/\mathrm{shoe}}={\left(\frac{1}{k_{\mathrm{shoe}}}+\frac{1}{k_{\mathrm{arch}}}+\frac{1}{k_{\mathrm{pMTU}}}\right)}^{-1} $$

where *k*_foot/shoe_ is the system stiffness of the foot-shoe interface, *k*_shoe_ is the MBS of a shoe, *k*_arch_ is the stiffness of the arch, and *k*_pMTU_ is the stiffness of the pMTU. It needs to be noted that cushioning stiffness and MBS of a shoe are two distinctive shoe properties that should not be used interchangeably; however, the findings of Kelly et al. [11] and the findings of this study suggest that by increasing either of these stiffnesses, similar increases in apparent arch stiffness can be observed.

### Limitations

There are some limitations associated with this study. The MTU models developed in this study represent approximations of biarticulated MTUs. For the sMTU model, the main purpose was to estimate its mechanics at its distal end because the ankle joint was suggested to be the main positive work generator of the lower limb during running [27]. It is possible, however, that the orientation of the joint at the proximal end of the sMTU (i.e. knee joint) could have influenced its mechanics, which was not accounted for in the model used in this study. Furthermore, the pMTU model represented a functional unit consisting of intrinsic foot muscles and the PA. It is likely, however, that the pMTU also included contribution from extrinsic foot muscles (e.g. tibialis posterior).

The methods used to estimate MTP/ankle [5, 6, 27] and arch [24] kinetics differed from each other. Three light-reflective markers were placed on the distal and proximal segment, respectively, of the MTP and ankle joint to measure segmental angular acceleration. For the arch, placing three markers on the distal (metatarsals) and proximal (calcaneus) segment, respectively, had required cutting additional holes in the shoe. This, however, could have compromised the structural integrity of the shoe. Because different methods were used to estimate MTP/ankle and arch joint kinetics, the interpretation of joint moments, powers, and work should focus on differences between footwear conditions instead of the differences between joints.

Similarly to Riddick et al. [24], the pMTU model used in this study is an estimate of unified PA and intrinsic foot muscle behaviour. Parsing out the contributions of individual structures of the foot cannot be done using this method. Electromyographic analyses of intrinsic foot muscle function have suggested that different muscles can show different activation patterns during the stance phase of walking and running [25]. Therefore, it is possible that some intrinsic foot muscles have shortened more than others. Although individual length changes and specific functions of various intrinsic foot muscles and the PA cannot be distinguished between in this study, it can be assumed that these structures of the foot act as a functional unit in response to the GRFs and arch deformation experienced during running [25].

We estimated the pMTU force as a function of AT force (i.e. sMTU force) and vertical GRF [36]. Other methods have been proposed to approximate the PA [48] or pMTU [24] forces in previous literature. Therefore, we also used the methods of Erdemir et al. [48] and Riddick et al. [24] to compute and compare pMTU forces. In brief, Erdemir et al. determined the PA force by a linear relationship between AT force and vertical GRF, similar to this study. Riddick et al., however, estimated the pMTU force by dividing the arch moment by the distance between the arch joint and the insertion of the pMTU on the calcaneus. When these methods were used to approximate pMTU forces for our data, peak pMTU forces were 35.20 ± 8.55 N·kg^−1^ and 127.04 ± 37.95 N·kg^−1^ for the control, and 34.46 ± 9.34 N·kg^−1^ and 118.61 ± 26.20 N·kg^−1^ for the stiff condition. There was no significant difference in peak pMTU force between footwear conditions using the methods of Erdemir et al. (*p* = 0.59, *d* = 0.03) or Riddick et al. (*p* = 0.41, *d* = 0.26). It needs to be noted, however, that absolute pMTU forces varied tremendously between methods. In general, estimated muscle or MTU forces are overestimated when using a musculoskeletal modelling approach [49]. In addition, the estimation of pMTU force by the methods of Riddick et al. [24] is strongly dependent on the location and trajectory of the arch joint centre. Therefore, it needs to be acknowledged that absolute values of pMTU forces are probably not correct; however, no matter what approach was chosen to estimate pMTU forces, the conclusions drawn in this study are regarding a change in pMTU forces as a function of footwear condition and thus, remain the same.

The stretch/shortening velocities of the pMTU and sMTU were based on time-derivation of estimated length changes and angular velocity transformations, respectively. None of the MTU velocities were based on measured length changes. Therefore, it cannot be guaranteed that the reported values are true representations of the MTU behaviour on a tissue level. Accordingly, the results reported in this study should be interpreted in the context of these limitations and it should be focused on comparisons of estimate values between footwear conditions. Future studies should try to address these limitations by using in vivo ultrasound imaging to better approximate the true stretch/shortening velocities of MTUs surrounding the foot-shoe interface [15].

## Conclusions

In conclusion, running in shoes with increased MBS resulted in less deformation of the arch and pMTU, and in slower shortening velocities of the pMTU and sMTU during late stance. Slower shortening velocities of MTUs led to reduced positive work performed by the compliances surrounding the foot-shoe interface. Based on the cost of generating force hypothesis [40, 41], it can be speculated that slower shortening velocities due to increased stance times could be related to lower metabolic rates of running if the reduced MTU shortening velocities are attributed to the muscle. If the slower shortening velocities are attributed to the tendon, however, it could be indicative of reduced energy return capacities of the tendon. Future studies should determine if the observed changes in shortening velocities are due to changes in muscle or tendon behaviour to further elucidate the effects of MBS on the energetics of running.

## Data Availability

The datasets generated and/or analysed during the current study are available from the corresponding author on reasonable request.

## Abbreviations

3D: Three-dimensional
AA: Arch angle
Arc_pMTU_: Arc length
AT: Achilles tendon
COP: Centre of pressure
F_pMTU_: Plantar muscle-tendon unit force
F_sMTU_: Shank muscle-tendon unit force
GRF: Ground reaction forces
GT: Great toe
k_arch_: Arch stiffness
k_foot/shoe_: Stiffness of foot/shoe interface
k_shoe_: Shoe stiffness
MA_sMTU_: Shank muscle-tendon unit moment arm
MBS: Midsole bending stiffness
MH: Medial heel
MP1: Distal head of first metatarsal
MTP: Metatarsophalangeal
NT: Navicular tuberosity
PA: Plantar aponeurosis
pMTU: Plantar muscle-tendon unit
sMTU: Shank muscle-tendon unit
v_linear_: Linear velocity on the proximal end of the foot segment
v_pMTU_: Plantar muscle-tendon unit velocity
v_sMTU_: Shank muscle-tendon unit velocity
ω_ankle_: Ankle joint angular velocity
